# Supplementary material for: Identification of photoperiod-induced specific miRNAs in the adrenal glands of Sunite sheep (Ovis aries)
Source: Front Vet Sci. 2022 Jul 22;9:888207. doi: 10.3389/fvets.2022.888207 (PMC9354845; doi:10.3389/fvets.2022.888207)
Supplement: Supplementary file 1 [file Data_Sheet_1.ZIP › Supplementary file/miRDeep2 file.zip!/miRDeep2 file/Know_Structure/LP42A1/oar-mir-3955.pdf]

miRBase precursor : oar-mir-3955
Total read count : 309
oar-miR-3955-5p read count : 281
oar-miR-3955-3p read count : 12
remaining reads : 16

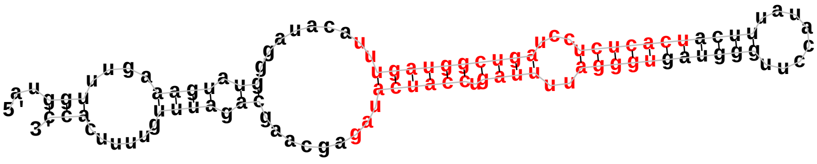

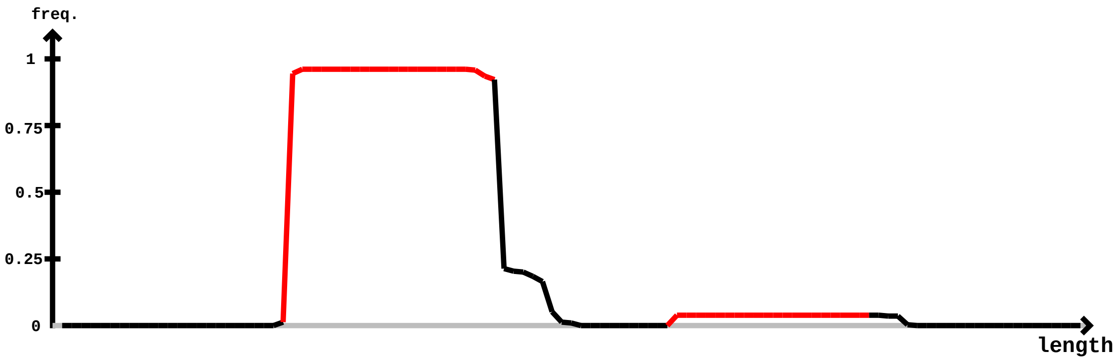


```
                                  oar-miR-3955-5p
                                                                        oar-miR-3955-3p
5'- augguuugaaaguaugggauacauuugauggcugauccucucacuacuuuauaccuuggguagugggauuuuaguccaucauagagcaagcagauuuguuuucacc   -3'    exp
    ...........((((...))))...((((((((((...(((((((((((........)))))))))))..)))).)))))).(((((((......)))))))....   reads   mm   sample
    ......................auuugauggcugauccucuca...............................................................   1       0    seq
    ......................auuugauggcugauccucucacu.............................................................   2       0    seq
    ......................auuugauggcugauccucucacuacuu.........................................................   1       0    seq
    .......................uuugauggcugauccucuc................................................................   1       0    seq
    .......................uuugauggcugauccucuca...............................................................   6       0    seq
    .......................uuugauggcugauccucucac..............................................................   4       0    seq
    .......................uuugauggcugauccucucacu.............................................................   215     0    seq
    .......................uuugauggcugauccucucacua............................................................   2       0    seq
    .......................uuugauggcugauccucucacuac...........................................................   1       0    seq
    .......................uuugauggcugauccucucacuacu..........................................................   4       0    seq
    .......................uuugauggcugauccucucacuacuu.........................................................   5       0    seq
    .......................uuugauggcugauccucucacuacuuu........................................................   34      0    seq
    .......................uuugauggcugauccucucacuacuuua.......................................................   12      0    seq
    .......................uuugauggcugauccucucacuacuuuau......................................................   1       0    seq
    .......................uuugauggcugauccucucacuacuuuaua.....................................................   3       0    seq
    ........................uugauggcugauccucucacu.............................................................   2       0    seq
    ........................uugauggcugauccucucacua............................................................   1       0    seq
    ........................uugauggcugauccucucacuacu..........................................................   1       0    seq
    ........................uugauggcugauccucucacuacuuu........................................................   1       0    seq
    ...............................................................ugggauuuuaguccaucauaga.....................   1       0    seq
    ...............................................................ugggauuuuaguccaucauagagc...................   10      0    seq
    ...............................................................ugggauuuuaguccaucauagagca..................   1       0    seq
```
